# Simultaneous occurrence of acute aortic dissection and acute infective endocarditis in a patient with chronic kidney disease

**DOI:** 10.1093/jscr/rjaf232

**Published:** 2025-04-19

**Authors:** Majd Hanna, Nafiza Martini, M H D Ghazi Aboulkher, Ahmad Walid Izzat, Mohammad Bashar Izzat

**Affiliations:** Damascus University, Faculty of Medicine, Damascus, Syrian Arab Republic; Stemosis for Scientific Research, Damascus, Syrian Arab Republic; Damascus University, Faculty of Medicine, Damascus, Syrian Arab Republic; Stemosis for Scientific Research, Damascus, Syrian Arab Republic; Damascus University, Faculty of Medicine, Damascus, Syrian Arab Republic; Stemosis for Scientific Research, Damascus, Syrian Arab Republic; Damascus University, Faculty of Medicine, Damascus, Syrian Arab Republic; Damascus University, Faculty of Medicine, Damascus, Syrian Arab Republic

**Keywords:** aortic dissection, infective endocarditis, chronic kidney disease, simultaneous occurrence, case report

## Abstract

Chronic kidney disease increases risk of cardiac complications. Concurrent aortic dissection and infective endocarditis is exceptionally rare. A 29-year-old male with hypertension and chronic kidney disease post-renal transplant presented with chest and back pain. Imaging revealed acute Stanford Type A aortic dissection. Emergency surgery also uncovered undiagnosed infective endocarditis. The patient underwent aortic root replacement and was treated with intravenous antibiotics for 6 weeks postoperatively. He had an uneventful recovery without cardiac or infective complications. Physicians should maintain a high index of suspicion for concurrent cardiac complications in symptomatic chronic kidney disease patients, as prompt diagnosis and treatment is crucial for good outcomes in these rare cases.

## Introduction

Chronic kidney disease has been reported to be associated with a number of cardiac surgical complications; including aortic dissection, valvular disease, and myocardial ischemia. While it is uncommon for one patient to suffer from multiple cardiac surgical complications at the same time, sporadic instances of simultaneous occurrence of multiple cardiac surgical complications have been documented infrequently in the medical literature.

We are reporting a case of a patient with chronic kidney disease who underwent emergency surgery for acute aortic dissection. During the surgical procedure, it was discovered that he had undiagnosed acute infective endocarditis of the aortic valve. Understanding this infrequent coexistence of simultaneous cardiac surgical complications serves as a critical reminder to treating physicians to remain vigilant about this possibility.

## Case history and management

A 29-year-old male who had been experiencing high-grade fever for several days was admitted to the emergency department following the onset of acute and severe chest and back pain. In his past medical history, he was known to suffer from hypertension-induced chronic kidney failure, a prior renal transplantation 10 years earlier with an immune rejection and return to dialysis dependence for 5 years. Emergency room electrocardiogram revealed normal sinus rhythm with signs of left ventricular hypertrophy, and laboratory investigations unveiled leucocytosis (15 000/μl) and anemia (3.24·106/μl). All other serological and hematological parameters were within normal ranges. Emergency room transthoracic echocardiography revealed the presence of a dilated ascending aorta, severe aortic valve incompetence with mild pericardial effusion. Emergency chest and abdominal contrast computed tomographic scanning revealed a Stanford type A aortic dissection; hence, decision was made to proceed with immediate surgery to replace the aortic root (Bentall procedure).

During the surgical procedure, the characteristic signs of ascending aortic dissection were present, however inspection of the aortic valve disclosed the presence of large vegetations on aortic valve cusps indicative of acute infectious endocarditis. Immediate treatment with wide-spectrum antibiotics was initiated intraoperatively, comprising piperacillin (4 gm) and tazobactam (0.5 gm) every 6 h, complemented by ciprofloxacin (200 mg) every 12 h. This therapy was continued postoperatively based on the results of bacteriology which was positive for *Staphylococcus aureus*. Aortic root replacement was carried out, and there were no cardiac complications postoperatively; however, renal function remained compromised, necessitating the use of continuous veno-venous hemofiltration. After a span of 5 days, the patient was successfully weaned off the ventilator and extubated. Postoperative course was uneventful, and there were no other cardiac or infective complications. The patient was referred to continue his medical treatment under the care of the nephrologists.

## Discussion

The concurrent existence of acute aortic dissection and infective endocarditis is an exceedingly rare occurrence, with a very limited number of documented cases in the medical literature [1–4]. Several key factors are likely to have predisposed to this uncommon association in this patient, namely hypertension, chronic kidney disease, immunosuppressants, and hemodialysis, which can all increase tissue fragility as well as susceptibility to infection.

Long-standing hypertension is known to induce altered hemodynamic stress to the aortic wall and direct damage to the aortic media and intimal thickening, thereby increasing susceptibility to aortic dissection [5]. Moreover, accumulating evidence indicates that chronic kidney disease contributes to the progression of atherosclerosis and cardiac dysfunction. This phenomenon is mediated by various mechanisms including endothelial dysfunction, volume expansion, cytokine secretion, sympathetic nervous system activation, anemia, inflammation, and activation of the renin-angiotensin-aldosterone system [6–9].

This patient's history of renal transplantation is likely to have increased his susceptibility to developing infective endocarditis, primarily due to prolonged exposure to corticosteroids, hemodialysis, and intensive immunosuppressive regimens [10]. Importantly, intensified steroid therapy and the escalation of immunosuppression to treat renal allograft rejection may have further elevated the risk of infective endocarditis [10].

Both type A aortic dissection and infective endocarditis are critical conditions that necessitate prompt diagnosis and treatment due to their significant complications and unfavorable outcomes. Fortunately, due to recent advancements and increased awareness, there has been a notable reduction in mortality associated with type A aortic dissection, decreasing from ~31% to 22% [11]. On the other hand, the mortality rate of infective endocarditis in transplant recipients has been reported to range from 44% to 57% [12, 13]. Fortunately, through timely and appropriate management, our patient achieved a successful outcome.

## Conclusion

Though exceptionally rare, treating physicians must remain highly suspicious about the possibility of the simultaneous coexistence of multiple cardiac surgical complications in symptomatic patients with chronic kidney disease and/or kidney transplant, as expedited diagnosis and swift intervention are imperative in achieving satisfactory outcomes.
